# Frequency of retinopathy of prematurity (ROP) in infants screened for ROP: two years follow-up results of a single center in Turkey

**DOI:** 10.37796/2211-8039.1188

**Published:** 2021-09-01

**Authors:** Adem Ugurlu

**Affiliations:** Erzincan Binali Yildirim University, Faculty of Medicine, Department of Ophthalmology, 24100, Erzincan, Turkey

**Keywords:** Retinopathy of prematurity, Screening, Frequency

## Abstract

**Aim:**

The aim of this retrospective study was to determine the incidence of retinopathy of prematurity (ROP) in infants referred to our clinic for screening ROP.

**Material and method:**

The data of 729 infants who were referred to the ROP outpatient clinic of the Ophthalmology Unit of Erzincan Binali Yildirim University, Turkey between April 2018 and March 2020 were analyzed retrospectively. The gestational age and weight of the infants, stay in the neonatal intensive care unit, duration of oxygen therapy, and detailed ophthalmologic examination findings were recorded in the study.

**Findings and results:**

Of the 729 babies screened for ROP, 122 (16.7%) of them had ROP. Infants with gestational age of ≤28 weeks constitute 3.3% of all infants and ROP rate was significantly higher than infants with older gestational age (*P* < 0.001). There were 39 babies born under 1000 grams and ROP was present in 28 (71.8%) of these infants and the incidence of ROP was higher than infants with higher birth weight (*P* < 0.001). With the result of logistic regression analysis, gestational age (OR:0.592,95% CI:0.208–0.779,*P* < 0.001), stay in NICU (OR:0.998,95% CI:1.022–1.421,*P* = 0.001), and duration of oxygen (O_2_) therapy (OR:34.309, 95% CI:2.043–28.235,*P* = 0.004) were detected the independent risk factors for ROP.

**Conclusion:**

Although infants with ≤1000 grams gestational weight and ≤28 weeks gestational age are more likely to have ROP, it is clear that screening for all infants at risk, regardless of gestational weight and age, is very important in preventing ROP-related vision loss. In addition, it is also recommended to control the duration of staying in neonatal intensive care unit and oxygen therapy to as little as needed.

## Background

Retinopathy of prematurity (ROP) is the name given to vasoproliferative retinal disorder that occurs in the developing retinas of premature neonates.[1,2] The development of retinal vessels in the fetus begins at the 16th gestational week.[3–5] This vascular development, which starts from the optic nerve head and progresses to the peripheral retina, reaches the nasal ora serrata at the 36th gestational week and the temporal ora serrata at the 40th gestational week.[6,7] Therefore, in premature babies, the retina is not fully vascularized at birth, and peripheral avascular zone may be present in the retina in relation to the gestational week at birth.[8–11] ROP is the most common cause of preventable blindness in infants.[12,13] Therefore, as in the whole world, great importance is attached to the screening of premature babies in Turkey.[14,15].

In our study, it was aimed to investigate the frequency of ROP among babies who were born prematurely in the last 2 years and were referred to our ophthalmology department for ROP screening.

## Material and method

This study was a single centre, cross-sectional, hospital-based review of medical records of patients referred to the Erzincan Binali Yildirim University Mengucek Gazi Training and Research Hospital Ophthalmology Unit ROP outpatient clinic for screening was included in the study from April 2018 to March 2020. The data of 769 babies examined between April 2018 and March 2020 were analyzed retrospectively. Forty babies were excluded the study due to the loss to follow-up. Verbally informed consent was obtained from the parents of all infants. The study was carried out by adhering to the Helsinki Declaration principles. The gestational age and weight of the infants, stay in the neonatal intensive care unit, duration of oxygen therapy, and detailed ophthalmologic examination findings were recorded in the study. All of the babies included in the study were taken non-invasive mechanical ventilation.

### ROP screening procedure

Babies smaller than 37 weeks of gestational age were examined for ROP. All babies were examined at 4–6 weeks after birth. ROP screening was performed after applying tropicamide 1% (Tropamid^®^, Bilimilaç^®^,Gebze, Turkey) and phenylephrine hydrochloride 0.5% (Mydfrin^®^, Alcon^®^, Fort Worth, TX) eye drops three times for pupillary dilatation. ROP screening was performed by an experienced ophthalmologist using binocular indirect ophthalmoscope combined with a scleral depressor after applying proparacaine hydrochloride 0.5% (Alcaine^®^, Alcon^®^, Fort Worth, TX) eye drops as the topical anesthetic. The grading of the ROP status was made according to the international ROP classification.[ 1] For each infant, the status of ROP was recorded including the zone, stage, extent of the disease, and the presence or absence of plus disease in the study.[8,10,12] After ROP screening procedure, each infant was also graded according to the maximum stage of ROP in both eyes. Subjects that were not diagnosed with ROP were followed up every two to three weeks until retinal vascular maturation was completed. Subjects diagnosed with ROP were followed up every one to two weeks due to the severeness of the disease.

### Statistical analysis

IBM SPSS (Statistical Package for the Social Sciences) 22.0 package program was used to perform statistical analysis. The average, standard deviation, percentage, minimum and maximum values of the data were calculated. Chi-square test was used in the analysis of categorical data. The normal distribution of data was calculated using the Kolmogorov-Smirnov test. Pearson correlation analysis was used for normally distributed data for correlation analysis. If there is no normal distribution, Spearmann correlation analysis was used for the analysis of the data. Continuous variables were expressed as mean ± standard deviation (SD), and categorical variables as frequencies and percentages. The Student's t-test was performed for intergroup comparisons of the continuous variables. In addition, the Analysis of Variation (ANOVA) test for homogenous data and the Mann-Whitney U test for non-homogenous data were used for statistical analysis. To predict the significant independent risk factors associated with the existence of ROP, logistic regression analysis was performed in the study. The adjusted odds ratio (OR) and the 95% confidence interval (CI) for each possible risk factor were calculated. Exact *P* values of <0.05 were accepted statistically significant for the analyzes.

## Results

Of the 729 babies included in the study, 372 were male and 357 were female. The mean gestational age of infants was 33.4 ± 2.3. The mean gestational weights of the babies were 2120.6 ± 231.9 grams. Among the 729 babies screened, the number of babies with ROP was 122 (16.7%). There were 7 (0.9%) babies underwent ROP treatment (4 intravitreal anti-VEGF and 3 laser photocoagulation therapy) in the study. In infants diagnosed with ROP, the most advanced stage of ROP was evaluated and recorded. Aggressive posterior ROP was detected in 1 (0.8%) infant. While 18 (14.8%) infants were stage 3, 42 (34.4%) infants were stage 2 and 61 (50%) infants in stage 1. When the ROP was first diagnosed in patients, 16 (13.1%) infants were zone 1, 88 (72.1%) infants were zone 2 and 18 (14.8%) infants were zone 3 ROP. Demographic and clinical features of the patients are shown in Table 1. The incidence of ROP in infants according to their gestational age and weight are shown in Tables 2 and 3, respectively.

The logistic regression analysis was performed to determine the independent risk factors for ROP. According to the results of this analysis, gestational age (OR: 0.592, 95% CI: 0.208–0.779, *P* < 0.001), stay in NICU (OR: 0.998, 95% CI: 1.022–1.421, *P* = 0.001), and duration of oxygen therapy (OR: 34.309, 95% CI: 2.043–28.235, *P* = 0.004) were detected as the independent risk factors for ROP (Table 4).

## Discussion

Since the diagnosis and treatment of ROP requires serious clinical experience, it poses certain difficulties for ophthalmologists.[16–19] Today, ROP is detected before the serious stages with the increasing awareness of pediatricians and parents and in cases where treatment is required, intervention is made without delay.[20–23] In the treatment of ROP, the location and stage of the disease in the retina is the most important factor in determining the treatment option.[24–28] In the presence of ROP requiring treatment close to the posterior pole, intravitreal anti-VEGF agents are considered in the foreground, whereas when a ROP case requiring treatment with peripheral avascular retinal areas farther away from the posterior is present, laser photocoagulation option is considered in the foreground.[24,29–31].

Bas et al. tried to determine the ROP incidence, risk factors and severity with the prospective TR-ROP study.[32] In this study, which included 6115 infants, it was concluded that the screening of babies with a gestational age of ≤34 weeks and a birth weight of ≤1700 grams was suitable for our country. Our study also showed that babies with a gestational age of ≤35 weeks and a gestational weight of ≤2000 grams are at greater risk of developing ROP. However, risk factors such as systemic diseases, blood transfusion and sepsis couldn't be evaluated enough in our study and it makes the comparison difficult with the study. The frequency of ROP varies according to the development levels of the countries and the features of the neonatal intensive care units. In developed countries, ROP is predominantly a problem of preterms born below 28 weeks, while in developing countries it is reported that severe ROP develops up to 34 weeks.[33–37].

In the multicentre study conducted by the Turkish Neonatology Association in 2014, the frequency of ROP was 42% and the frequency of advanced ROP was 8.2% in preterm babies with very low birth weight (<1500 grams). In this study, the frequency of ROP in infants older than 32 weeks of gestation was 13.3% and advanced ROP was 0.4% in these infants. Advanced ROP was found in 20 babies over 32 weeks of gestational age, 41 babies with >1500 grams gestational weight and 3 babies with >2000 grams gestational weight. The results of the study showed that advanced ROP requiring treatment may develop in infants with higher gestational age and weight.[38].

Larsen et al. evaluated the incidence of retinopathy of prematurity in Germany.[39] They stated that in their investigated cohorts, preterm infants with higher GA than 30 weeks carried a very low or no risk for developing treatment-requiring ROP unless additional risk factors were present, and no treatment was performed earlier postmenstrual age than 32 weeks. And also they pointed out that these findings are of relevance for the ongoing re-evaluation of ROP screening criteria.

In our study, the number of ROP with advanced stage and requiring treatment are only 7 and 4 of these babies underwent laser photocoagulation and 3 for intravitreal anti - VEGF treatment. All of these infants were with ≤28 weeks of gestational age and ≤1000 grams of gestational weight. The reason for the low number of advanced ROP babies requiring treatment in our clinic is that our neonatal intensive care unit is not an advanced treatment unit and these babies in the risky group (having systemic infections, blood transfusion, sepsis, etc.) are directed to centers with advanced neonatal intensive care units. This is the major limitation of our study.

Some of the babies referred to our clinic are between 35–37 weeks of gestation, and are screened for ROP. A small proportion of these babies have ROP which is not requiring treatment, and this was an important finding to show that ROP may be seen in babies with over 35 weeks of gestation. ROP screening is also important, especially in infants who have higher gestational age and weight but have accompanying systemic disease.[40] There weren't any big babies especially with systemic risk factors such as sepsis, blood transfusion, etc. In our study and this is a limitation to compare the studies.

## Conclusion

ROP screening and treatment, especially for babies with lower gestational age and weight will provide great gains in combating this disease, which is the most common cause of preventable blindness in infants. In addition, it is also recommended to control the infants for ROP while staying in neonatal intensive care unit and make the oxygen therapy as little as needed.

## Figures and Tables

**Table 1:** Demographic and clinical features of the patients.

	ROP	No ROP	Total	P value
Number of patients	122 (16.7%)	607 (83.3%)	729 (100%)	<0.001
Gender(M/F)	63(51.6%)/59(48.4%)	309(50.9%)/298(49.1%)	372(51%)/357(49%)	0.879
GA (weeks)	30.4 ± 2.2	34.7 ± 1.9	33.4 ± 2.3	0.003
GW (grams)	1947.2 ± 198.4	2199.7 ± 251.2	2120.6 ± 231.9	<0.001
Stay in NICU	29.8 ± 3.7 days	5.91 ± 2.8 days	8.93 ± 4.56 days	<0.001
Duration O_2_ therapy	18.6 ± 4.5 days	2.4 ± 1.5 days	4.72 ± 3.57 days	<0.001

GA:Gestational age GW:Gestational weight NICU:Neonatal intensive care unit M:male F:female.

**Table 2:** The incidence of ROP in our patients according to their gestational age.

Gestational age	Total n (%)	ROP n (%)	No ROP n (%)	P value*
≤28 weeks	24 (3.3%)	17 (70.8%)	7 (29.2%)	<0.001
29–31 weeks	129 (17.7%)	36 (27.9%)	93 (72.1%)	<0.001
32–35 weeks	488 (66.9%)	64 (13.1%)	424 (86.9%)	<0.001
≥35 weeks	88 (12.1%)	5 (5.7%)	83 (94.3%)	<0.001
Total	729 (100%)	122 (16.7%)	607 (83.3%)	

* Difference between the infants with and without ROP.

**Table 3:** The incidence of ROP in our patients according to their gestational weights.

Gestational weight	Total n (%)	ROP n (%)	No ROP n (%)	P value*
≤1000 grams	39 (4.5%)	28 (71.8%)	11 (28.2%)	<0.001
1001–1500 grams	138 (18.9%)	38 (27.5%)	110 (72.5%)	<0.001
1501–2000 grams	167 (23.2%)	24 (14.4%)	149 (85.6%)	<0.001
2001–2500 grams	199 (27%)	21 (10.6%)	178 (89.4%)	<0.001
≥2500 grams	186 (26.3%)	11 (5.9%)	175 (94.1%)	<0.001
Total	729 (100%)	122 (16.7%)	607 (83.3%)	

* Difference between the infants with and without ROP.

**Table 4:** Logistic regression analysis for independent risk factors of ROP.

Variable	OR	95% CI	P value
GW	0.934	0.976–1.521	0.381
GA	0.592	0.208–0.779	<0.001
Sex	0.685	0.374–2.129	0.379
Stay in NICU	0.998	1.022–1.421	0.001
Oxygen removal time	34.309	2.043–28.235	0.004

GA: Gestational age GW: Gestational weight NICU: Neonatal intensive care unit.
